# Performance Enhancement of Land Vehicle Positioning Using Multiple GPS Receivers in an Urban Area

**DOI:** 10.3390/s16101688

**Published:** 2016-10-14

**Authors:** Jong-Hwa Song, Gyu-In Jee

**Affiliations:** 1 AESA Radar R&D Center, Hanwha Systems, Gyeonggi-do 17121, Korea; jh.song@hanwha.com; 2 Department of Electronics Engineering, Konkuk University, Seoul 05029, Korea

**Keywords:** multiple GPS receivers, RAIM, outlier, closeness, double-difference, urban

## Abstract

The Global Positioning System (GPS) is the most widely used navigation system in land vehicle applications. In urban areas, the GPS suffers from insufficient signal strength, multipath propagation and non-line-of-sight (NLOS) errors, so it thus becomes difficult to obtain accurate and reliable position information. In this paper, an integration algorithm for multiple receivers is proposed to enhance the positioning performance of GPS for land vehicles in urban areas. The pseudoranges of multiple receivers are integrated based on a tightly coupled approach, and erroneous measurements are detected by testing the closeness of the pseudoranges. In order to fairly compare the pseudoranges, GPS errors and terms arising due to the differences between the positions of the receivers need to be compensated. The double-difference technique is used to eliminate GPS errors in the pseudoranges, and the geometrical distance is corrected by projecting the baseline vector between pairs of receivers. In order to test and analyze the proposed algorithm, an experiment involving live data was performed. The positioning performance of the algorithm was compared with that of the receiver autonomous integrity monitoring (RAIM)-based integration algorithm for multiple receivers. The test results showed that the proposed algorithm yields more accurate position information in urban areas.

## 1. Introduction

At present, the Global Positioning System (GPS) is the most widely used sensor for land vehicle navigation systems, as it provides reasonable positioning solutions in terms of absolute coordinates at most locations and times worldwide and it is free of cost [1,2]. In urban areas, skyscrapers and other tall structures prevent the GPS signal propagation, which leads to degradation of the positioning performance due to poor measurement, multipath interference, and non-line-of-sight (NLOS) errors [3,4].

Many techniques have been proposed to enhance the positioning accuracy, availability and reliability of the GPS in urban areas by mitigating and removing the effects of multipath and NLOS errors. The common method involves integrating the GPS with another sensor. A representative sensor is the inertial measurement unit (IMU) because its characteristics complement those of the GPS. The IMU is incorporated in full or reduced order (dead reckoning) to consider the characteristics of load vehicle dynamics [2,5,6]. Another method involves detecting and excluding multipath or NLOS errors by checking the signal propagation path [7,8,9,10,11,12]. The omnidirectional infrared (IR) camera is used to eliminate invisible satellites by comparing the geometrical relation between the satellites and obstructions [7,8]. In [9], a Light Detection and Ranging (LiDAR) sensor was used to obtain the digital surface of the environment, and the results were used as an external source of information on expected reflections. On another study, by obtaining environmental information from real-time using sensors, a three-dimensional (3D) building model map was used to detect NLOS reception [10,11,12].

In yet another scheme, multipath errors can be mitigated by using only the GPS without additional sensors or prior information [13,14,15]. To estimate the pseudorange error, the urban canyon model using a ray tracing algorithm is presented [13]. In [14,15], raw data from a single GPS receiver, such as pseudorange and Doppler, were modeled based on a “contrario” to partition a dataset between inliers and outliers, and the classical particle filter (PF) was used to consider non-Gaussian noise. When a single GPS receiver is used, the number of observations can be reduced in urban areas, since the signal is blocked by tall buildings. Thus, system redundancy is reduced, and outlier detection performance degrades. In some cases however, even the outlier detection method cannot be applied due to the insufficient observations.

One method to enhance system redundancy is the use of multi-constellation observations. In [16], the GPS/Galileo observations were combined and erroneous measurements were detected using the receiver autonomous integrity monitoring (RAIM) algorithm in an urban area. The results showed that the use of multiple global navigation satellite systems (GNSSs) improves positioning performance in terms of accuracy and availability. However, these methods require a multi-constellation system and receivers. On the contrary, system redundancy can be improved by using multiple GPS receivers with a short baseline length (<2 m). The use of additional similar observations improves estimation accuracy by averaging the effect from different errors in observation. In [17,18], inexpensive multiple GPS receivers were integrated by simply averaging the calculated positions and incorporating raw measurements into them in open sky conditions. The results showed that position performance improved in terms of accuracy and availability. Furthermore, the performance of system integrity checks can be improved, because the additional measurements increase system redundancy. Therefore, the use of multiple receivers improves the detection capability of outliers, including multipath and NLOS errors. In [19], multiple GPS receivers were integrated to detect multipath errors in an urban area. In this method, the cross-correlation among antennas was computed to study the multipath spatial correlation properties among antennas on a moving platform. The results showed a weak correlation in the code-minus-carrier differences among antennas in both open sky and urban areas. It implies that the measurements obtained in urban areas from multiple receivers with a short baseline can have different errors. This can be caused by different error sizes and affection time from the same error source. Using this result, the multipath error was detected and removed based on the RAIM algorithm.

However, the above method encounters a problem when multiple outliers are observed because the detection performance of the RAIM algorithm degrades in the case of multiple outliers. In the conventional RAIM algorithm, position is calculated using all observations to predict input observations. The measured and predicted observations are then compared to detect outliers [20,21]. Hence, the effect of outliers is always included in the predicted observations. When multiple, significant outliers occur in an observation, the RAIM algorithm can fail to detect them, despite increased redundancy. Several methods have been proposed to solve multiple outlier problems. In [22], observations were iteratively checked for outliers following the rejection of one outlier. Moreover, all possible subsets of measurements were considered in detecting and rejecting multiple outliers in [23]. The drawbacks common to most proposed methods for multiple outliers are that they are computationally much more intensive than simple data snooping and, yet, do not yield dramatic performance improvement [20].

The limitation of the RAIM algorithm has prompted the development of new outlier detection algorithms. One possible approach involves detecting some outliers prior to applying the RAIM algorithm. Measurements by multiple receivers from the same satellite can be used to detect outliers by comparing the results of the receivers, since these measurements should be theoretically identical, except for noise, receiver clock error and geometrical differences, in open sky. When multipath or NLOS error is included in a measurement, its difference from other measurements increases. Thus, the outlier can be detected by comparing this difference. However, there is ambiguity in classification between correct and wrong measurements. In detail, the closed measurements do not always guarantee correct results since these measurements can have a common bias. To check whether the closed measurements are inliers, the mean SNR is calculated and tested. This is based on the fact that the multipath or NLOS measurements have lower SNR than direct ones [10]. In this paper, a new outlier detection method for multiple GPS receivers is proposed to overcome the limitations of the RAIM algorithm. This approach is based on the use of highly correlated measurements from the same satellite and the low cross correlation properties among measurements obtained in urban areas, as mentioned above [19].

The proposed outlier detection method consists of two stages. In the first stage, the closeness of the pseudoranges from the satellite is tested. Prior to the test, GPS-related terms not common to all pseudoranges are removed. In order to do so, the double-difference technique is applied. The difference in the positions of receivers is one cause of terms that are not common to pseudoranges. It can be calculated by projecting the known baseline vector in the direction of the line of sight (LOS), and this is subtracted from the double-difference pseudorange. Then, this value is tested to check the closeness, and outliers are detected. Finally, the SNR test is applied to distinguish the outliers that have a common bias. In the second stage of the proposed method, the remaining measurements are tested again by the RAIM algorithm. Due to the outlier detection in the first stage, the effect of outliers in the RAIM algorithm is reduced, and positioning performance improves.

The contribution of this paper is the development of an outlier detection algorithm that does not require calculating the position. Thus, the effects of outliers on other measurements are removed. The relationship between the position of multiple antennas was derived to integrate the measurements of multiple receivers. Moreover, the RAIM algorithm can be applied following the application of the proposed detection algorithm. Hence, better outlier detection performance can be achieved. A live test had been performed to test and analyze the proposed method in an urban area. The results showed the proposed method enhanced the position performance. Also, the advantages and drawbacks of the proposed methods are presented.

Four assumptions are made in the proposed method: (1) The baseline vector is defined as the relative vector between the reference point and the antenna position. This is obtained in advance by post-processing the carrier phase measurement of multiple receivers using the relative positioning method; (2) To calculate the double-difference pseudorange, we assume that there exists a reference satellite for which all pseudoranges of all relevant GPS receivers are inliers to reduce detection uncertainty. The measurements obtained from a satellite with a high elevation angle might not contain bias errora; thus, the assumption is reasonable. The reference satellite is selected by comparing the elevation angle with the signal-to-noise ratio (SNR); (3) The pitch and roll angles of the vehicle are ignored because these values are generally small in land vehicle applications; (4) For the sake of simplicity, the calculation of a vehicle’s heading angle is not considered in the proposed method. Instead, we assume that it can be calculated and obtained from other measurements, such as GPS Doppler and carrier phase, via well-known methods [24]. In this paper, the heading angle of the vehicle is obtained by adding noise to the value obtained from a reference sensor. The variance in the noise is selected by considering bad signal conditions [25].

This paper is organized as follows: the positioning algorithm for multiple receivers is presented in Section 2. This contains a description of the configuration of the overall system as well as the proposed positioning algorithm based on the iterative weighted least squares (IWLS) technique. The measurement model is obtained from the relationship between multiple receivers and the reference point. Section 3 is dedicated to the details of the proposed outlier detection and exclusion methods. The performance of the proposed approach is analyzed in comparison with the RAIM-based algorithm using live data in Section 4. Finally, the conclusions of this study and directions for future work in the area are explored in Section 5.

## 2. Positioning Algorithm for Multiple Receivers

**Figure 1:**
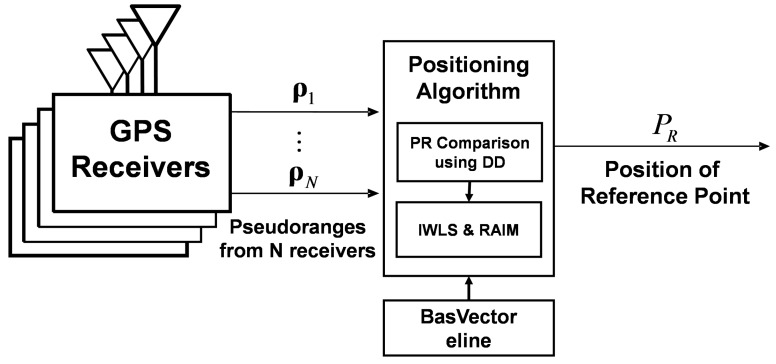
Block diagram of positioning system for multiple receivers.

Measurements from multiple receivers are integrated using a tightly coupled approach to estimate the position of the desired reference point in the vehicle. The tightly coupled approach means the integration of raw measurements, and where the proposed method integrates the pseudoranges from multiple receivers rather than positions. It is similar to the conventional GNSS/INS tightly coupled integration, which combines the raw measurements from two sensors. Figure 1 shows a block diagram of the proposed positioning system. The pseudoranges of each receiver are used as measurements and integrated using a geometrical relationship. In the positioning algorithm, the pseudorange measurements are first tested to detect the outliers, includeing multipath or NLOS errors. Pseudoranges from the same satellite are compared based on the double-difference technique. The passed and the suspended measurements in first test are inspected again using the conventional RAIM algorithm [20]. Finally, inlier measurements are integrated with one another to estimate the position of the reference point. The IWLS is selected as the estimator to evaluate the effect of the single epoch measurements. The details of measurement model and the IWLS are described in the remainder of this section.

### 2.1. Measurement Model

**Figure 2:**
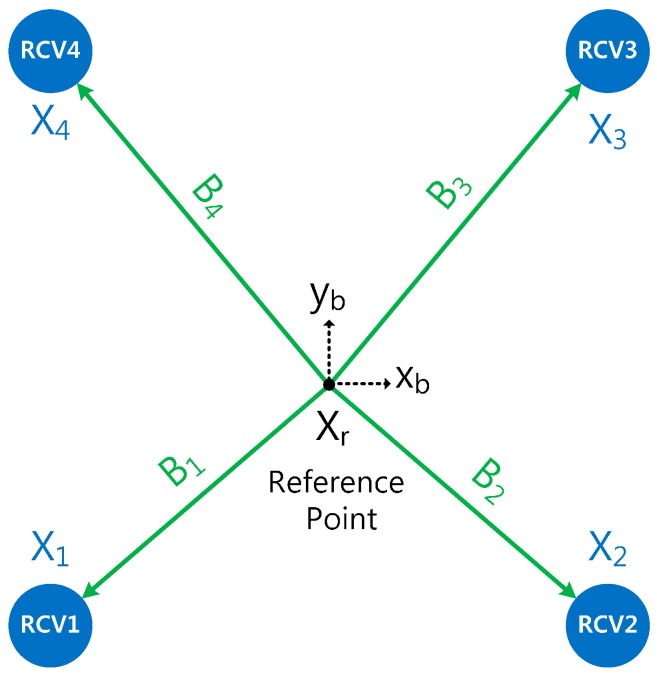
Relationship between four receivers and reference point.

In this paper, the position of the reference point is estimated instead of that of each receiver. To estimate this, the relationship between measurements from multiple receivers and the reference point need to be derived. Figure 2 shows the geometrical relationship between a reference point *X_r_* and the positions of four receivers *X_i_* in the navigation frame with a baseline vector *B_i_* given in the body frame. The position of each receiver is represented as a function of the baseline vector and the reference point using the geometrical relationship, can be expressed as in Equation (1):

(1) $$X_{i}=C_{b}^{n}B_{i}+X_{r}$$

where:

$X_{i}={\left[\begin{array}{ccc} n_{i} & e_{i} & d_{i} \end{array}\right]}^{T}$: position of multiple receivers in the navigation frame (*i* = 1, ..., *n*)
$X_{r}={\left[\begin{array}{ccc} n & e & d \end{array}\right]}^{T}$: position of reference point in the navigation frame
$B_{i}={\left[\begin{array}{ccc} x_{i}^{b} & y_{i}^{b} & z_{i}^{b} \end{array}\right]}^{T}$: baseline vector in the body frame
$\Theta ={\left[\begin{array}{ccc} \phi & \theta & \psi \end{array}\right]}^{T}$: attitude of vehicle in the reference point
$C_{b}^{n}$: rotation matrix from the body to the navigation frame
  $$C_{b}^{n}=\left[\begin{array}{ccc} \cos\theta \cos\psi & \sin\phi \sin\theta \cos\psi -\cos\phi \sin\psi & \cos\phi \sin\theta \cos\psi +\sin\phi \sin\psi \\ \cos\theta \sin\psi & \sin\phi \sin\theta \sin\psi +\cos\phi \cos\psi & \cos\phi \sin\theta \sin\psi -\sin\phi \cos\psi \\ -\sin\theta & \sin\phi \cos\theta & \cos\phi \cos\theta \end{array}\right]$$

**Figure 3:**
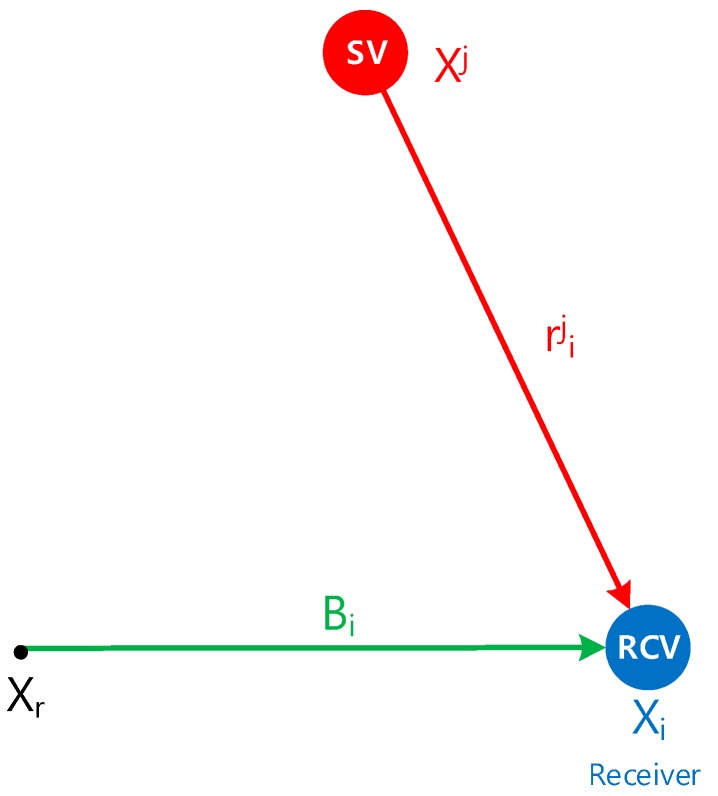
Relationship between range and reference point.

Following this, the relationship between the pseudorange of each receiver and the position of the reference point is described. The range of each receiver is a function of the satellite and the receiver’s position, and is defined as:

(2) $$r_{i}^{j}=\left\|X^{j}-X_{i}\right\|=\sqrt{{\left(n^{j}-n_{i}\right)}^{2}+{\left(e^{j}-e_{i}\right)}^{2}+{\left(d^{j}-d_{i}\right)}^{2}}$$

where $r_{i}^{j}$ is the geometrical range between satellite *j* and receiver *i*, and $X^{j}={\left[\begin{array}{ccc} n^{j} & e^{j} & d^{j} \end{array}\right]}^{T}$ is the position of satellite *j*. The range of each receiver can then be represented as a function of the reference point by substituting Equation (1) into Equation (2). The result is expressed as follows:

(3) $$\begin{array}{ll} r_{i}^{j} & =\left\|X^{j}-X_{i}\right\|=\left\|X^{j}-\left(X_{r}+C_{b}^{n}B_{i}\right)\right\|=\left\|X^{j}-\left(X_{r}+N_{i}\right)\right\|\\ & =\sqrt{{\left(n^{j}-\left(n+x_{i}^{n}\right)\right)}^{2}+{\left(e^{j}-\left(e+y_{i}^{n}\right)\right)}^{2}+{\left(d^{j}-\left(d+z_{i}^{n}\right)\right)}^{2}} \end{array}$$

where $N_{i}=C_{b}^{n}B_{i}={\left[\begin{array}{ccc} x_{i}^{n} & y_{i}^{n} & z_{i}^{n} \end{array}\right]}^{T}$ is the baseline vector of the receiver *i* in the navigation frame. Figure 3 shows the relationship between the range of each receiver and the reference point.

In Equation (3), the vehicle’s attitude is needed to transfer the baseline vector from the body to the navigation frame. As mentioned above, the roll and pitch angles are ignored. Therefore, the rotation matrix is calculated using only the heading angle.

### 2.2. Implementation of IWLS

The IWLS is implemented using the above results. Since the clock bias of each receiver is different, these should be estimated in order for measurements from multiple receivers to be used together. Thus, the state vector is defined as the reference point and clock bias of multiple receivers. The state variable and the measurement equation are represented by Equations (4)–(6):

- State variable:
  (4) $$X={\left[\begin{array}{ccccccc} n & e & d & b_{1} & b_{2} & \cdots & b_{N} \end{array}\right]}^{T}$$
  where, $b_{i}$ is the clock bias of the receiver and N is the number of receiver.
- Measurement equation:
  (5) $$z_{k}=h\left(X_{k}\right)+v_{k},\;v_{k}\sim N\left(0,R_{k}\right)$$
  where:
  $z_{k}={\left[\begin{array}{cccc} \rho_{1} & \rho_{2} & \cdots & \rho_{N} \end{array}\right]}^{T}$: pseudoranges of N receivers
  $\rho_{i}=\left[\begin{array}{cccc} \rho_{i}^{1} & \rho_{i}^{2} & \cdots & \rho_{i}^{m_{i}} \end{array}\right]$: pseudorange of *i*-th receiver
  $m_{i}$: the number of measured pseudoranges of *i*-th receiver
  $v_{k}={\left[\begin{array}{cccc} v_{1} & v_{2} & \cdots & v_{N} \end{array}\right]}^{T}$: measurement noise of N receivers
  $v_{i}=\left[\begin{array}{ccc} v_{i}^{1} & \cdots & v_{i}^{m_{i}} \end{array}\right]$: measurement noise of *i*-th receiver
  $h\left(X_{k}\right)={\left[\begin{array}{cccc} h_{1}\left(X_{k}\right) & h_{2}\left(X_{k}\right) & \cdots & h_{N}\left(X_{k}\right) \end{array}\right]}^{T}$: observation equation of N receivers
  $h_{i}\left(X_{k}\right)=\left[\begin{array}{cccc} h_{i}^{1}\left(X_{k}\right) & h_{i}^{2}\left(X_{k}\right) & \cdots & h_{i}^{m_{i}}\left(X_{k}\right) \end{array}\right]$: observation equation of *i*-th receiver
    $$\rho_{i}^{j}=h_{i}^{j}\left(X_{k}\right)+v_{i}^{j}\;=\sqrt{{\left(n^{j}-\left(n+x_{i}^{n}\right)\right)}^{2}+{\left(e^{j}-\left(e+y_{i}^{n}\right)\right)}^{2}+{\left(d^{j}-\left(d+z_{i}^{n}\right)\right)}^{2}}+b_{i}+v_{i}^{j}$$
- Linearization of measurement equation:
  (6) $$\begin{array}{ll} H_{k}=\frac{\partial h\left(X_{k}\right)}{\partial X_{k}} & ={\left[\begin{array}{cccc} \frac{\partial h_{1}\left(X_{k}\right)}{\partial X_{k}} & \frac{\partial h_{2}\left(X_{k}\right)}{\partial X_{k}} & \cdots & \frac{\partial h_{N}\left(X_{k}\right)}{\partial X_{k}} \end{array}\right]}^{T}\\ & ={\left[\begin{array}{cccc} H_{k,1} & H_{k,2} & \cdots & H_{k,N} \end{array}\right]}^{T} \end{array}$$
  where:
  $$H_{k,i}=\frac{\partial h_{i}\left(X_{k}\right)}{\partial X_{k}}==\left[\begin{array}{cccccccc} \frac{\partial \rho_{i}^{1}}{\partial n} & \frac{\partial \rho_{i}^{1}}{\partial e} & \frac{\partial \rho_{i}^{1}}{\partial d} & \underset{1\mathrm{st}}{0} & \underset{\cdots }{\cdots } & \underset{i\;\mathrm{th}}{1} & \underset{\cdots }{\cdots } & \underset{N\;\mathrm{th}}{0}\\ \frac{\partial \rho_{i}^{2}}{\partial n} & \frac{\partial \rho_{i}^{2}}{\partial e} & \frac{\partial \rho_{i}^{2}}{\partial d} & 0 & \cdots & 1 & \cdots & 0\\ \vdots & \vdots & \vdots & \vdots & \vdots & \vdots & \ddots & \vdots \\ \frac{\partial \rho_{i}^{m_{i}}}{\partial n} & \frac{\partial \rho_{i}^{m_{i}}}{\partial e} & \frac{\partial \rho_{i}^{m_{i}}}{\partial d} & 0 & \cdots & 1 & \cdots & 0 \end{array}\right]$$
- IWLS:

The IWLS is used to estimate position using a pre-defined state variable and a measurement equation. The state estimation and the update equation are shown in Equations (7) and (8):

(7) $$\Delta X_{k}={\left(H_{k}^{T}W_{k}H_{k}\right)}^{-1}H_{k}^{T}W_{k}\left(Z_{k}-{\hat{Z}}_{k}\right)$$

(8) $${\hat{X}}_{k}=X_{0}+\Delta X_{k}$$

where $X_{0}$ is the nominal state vector, $\Delta X_{k}$ is the error of nominal state vector, $W_{k}$ is the weighting matrix, and ${\hat{Z}}_{k}$ is the predicted measurement using the nominal state vector. The weighting factor of each measurement is inverse of variance $\sigma_{i}^{2}$, and is calculated using the SNR-based model represented in Equation (9) [21]:

(9) $$\sigma_{i}^{2}=A_{m}+B_{m}\times 10^{\left(-SNR_{i}\right)/10}$$

**Figure 4:**
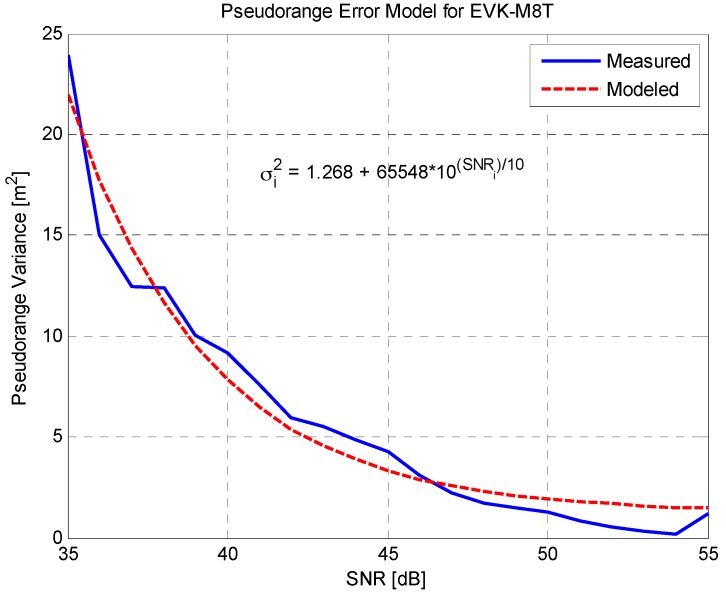
Pseudorange Error Model for EVK-M8T Receiver.

The model parameters $A_{m}$ and $B_{m}$ are derived by analyzing real data obtained in an open sky area for each receiver. The parameters are estimated by fitting the pseudorange errors to the above model using the LSE algorithm between valid SNRs, e.g., larger than 35 dB. The pseudorange errors are obtained from the reference sensor. Figure 4 shows the measured and modeled pseudorange error for the EVK-M8T receiver, which is one of the receivers used in this study and is mentioned in Section 4.1.

## 3. Outlier Detection and Exclusion for Multiple Receivers

In degraded signal environments, such as urban areas or forests, the detection and exclusion of outliers is difficult because of the insufficient number of measurements. Furthermore, large and multiple erroneous measurements degrade the capability of RAIM due to their mutual influence on measurements, in spite of the increased redundancy from multiple receivers. This paper proposes a two-stage outlier detection algorithm. In the first stage, the closeness of pseudoranges from the same satellites is checked without the mutual influence of measurements based on the double-difference technique. In the second stage, the RAIM algorithm is applied to remove the remaining outliers for each receiver measurement. The rejection of outliers in the first stage improves the detection ability of RAIM as well as the overall positioning performance. In the remainder of this section, the RAIM algorithm for multiple receivers used in this paper is described. Then, the proposed outlier detection method is explained.

### 3.1. RAIM Algorithm for Multiple Receivers

**Figure 5:**
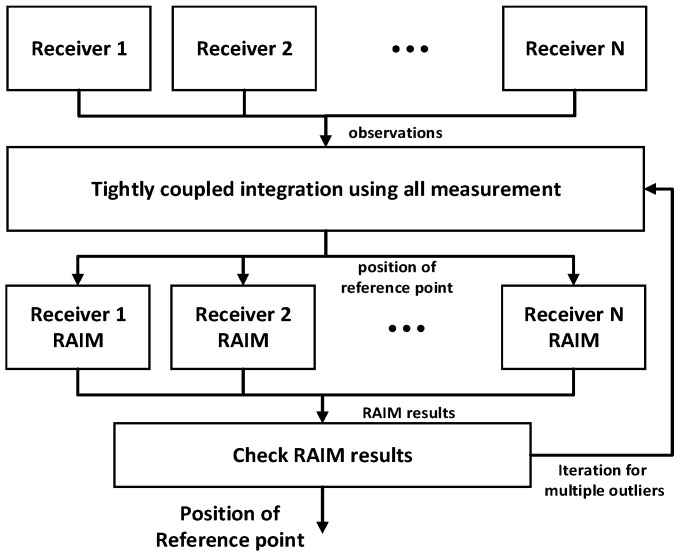
RAIM-based outlier detection for multiple receivers.

In the conventional RAIM algorithm, position is estimated using all measurements, and is used to calculate the measurement residual. A statistical test is then performed to detect outliers in measurements. Therefore, the more accurate the calculated position, the better the detection capability. In [19], the fixed constraints among multiple receivers are used as additional measurements to correct the positions obtained from multiple receivers. The RAIM algorithm is then applied to each receiver using the more accurate position. In this paper, the fixed constraints, i.e., the baseline vector, are utilized to integrate the pseudoranges of multiple receivers rather than their measurement. Figure 5 shows the RAIM-based outlier detection method for multiple receivers. First, pseudoranges from multiple receivers are combined in a tightly coupled approach using IWLS. Second, the RAIM algorithm is applied to observations of each receiver using the estimated position of the reference point. Finally, the RAIM results are checked and, if outliers exist, the above procedure is repeated until no additional outliers are left. In this approach, the integration of multiple receivers improves system redundancy and accuracy. For this reason, outlier detection capability is also enhanced. The detail RAIM algorithm used in this paper are described in [20].

### 3.2. Pseudorange Comparison Method

The measurements from multiples receivers with short baselines are highly correlated, and the differences among them are small. If some measurements include multipath or NLOS errors, the differences among measurements increase. Using this property, the pseudoranges of multiple receivers from the same satellite are compared and tested without calculating the position.

**Figure 6:**
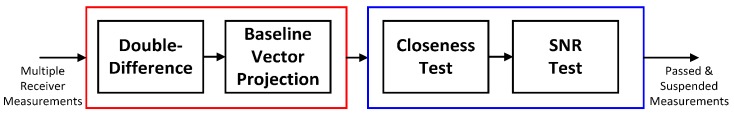
The block diagram of pseudorange comparison method.

The closeness of the pseudoranges from same satellite is inspected to detect outliers. Since measurements from multiple receivers contain the effects of different components, such as receiver clock and receiver position, these should be removed before the closeness test. Thus, the measured pseudoranges are pre-processed using the double-difference technique and baseline vector projection. The pre-processing results are then compared to detect outliers. Sometimes, there are many outliers, and they can contain a common bias error. In such cases, inliers can be removed instead of the outliers. To avoid this case from obtaining, an SNR test is performed for closed measurements. Unfortunately, when certain pseudoranges from multiple receivers from the same satellite are not measured, the proposed method cannot be applied to the satellite’s measurements, and a judgment cannot be made concerning outliers. In any case, the passed and the suspended measurements are sent to the RAIM algorithm to detect the remaining outliers. Figure 6 shows a block diagram of the pseudorange comparison method. In this figure, the red box represents the pre-processing block and the blue box the outlier detection and rejection test block. The details of each block are represented in the following.

Pre-processing is performed to reduce the difference among the pseudoranges of multiple receivers. The double-difference technique is first used to eliminate the difference due to GPS errors, especially receiver clock error. The double-difference pseudorange consists of range differences between the satellite and a given receiver, bias errors, and noise, and is represented by Equation (10):

(10) $$\rho_{AB}^{jk}=\rho_{AB}^{k}-\rho_{AB}^{j}=\left(r_{B}^{k}-r_{A}^{k}\right)-\left(r_{B}^{j}-r_{A}^{j}\right)+\left(M_{B}^{k}-M_{A}^{k}\right)-\left(M_{B}^{j}-M_{A}^{j}\right)+\varepsilon_{\rho_{AB}^{jk}}$$

where:

$\rho_{AB}^{jk}$: double-difference pseudorange for satellites *j* and *k* in Receivers A and B, respectively
$\rho_{AB}^{j}$: single difference pseudorange for satellite *j* in Receivers A and B
$r_{A}^{j}$: geometric range between satellite *j* and Receiver A
$M_{A}^{j}$: bias error included in pseudorange from satellite *j* to Receiver A
$\varepsilon_{\rho_{AB}^{jk}}$: noise in double-difference pseudorange

The reference satellite is chosen using the following conditions and process: first, the elevation angle of the satellite needs to be greater than 50°; second, pseudoranges are observed in all receiver; finally, find the satellite with the maximum mean SNR. If satellite *j* is assumed to be the reference satellite, bias errors of pseudoranges from satellite *j* are removed, and the Equation (10) is reduced to:

(11) $$\rho_{AB}^{jk}=\left(r_{B}^{k}-r_{A}^{k}\right)-\left(r_{B}^{j}-r_{A}^{j}\right)+\left(M_{B}^{k}-M_{A}^{k}\right)+\varepsilon_{\rho_{AB}^{jk}}$$

**Figure 7:**
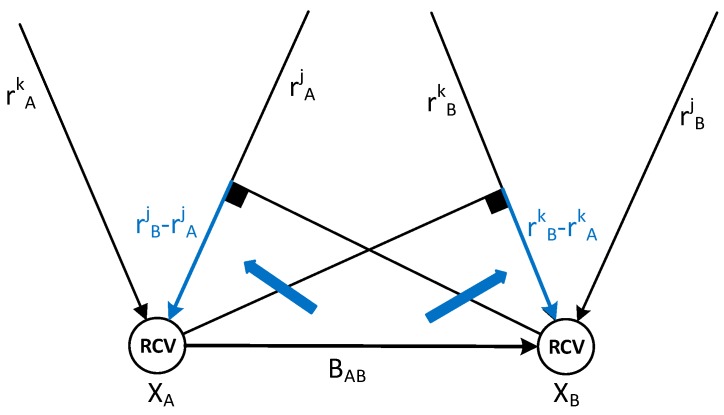
Relationship between range difference and baseline vector.

Furthermore, the geometrical distance among multiple receivers causes range differences in the double-difference pseudorange. The first and second terms of the right-hand side of Equation (11) represent range differences, which can be theoretically calculated by projecting the baseline vector in the direction of the LOS. Hence, range difference is reduced by subtracting the calculated value from the double-difference pseudoranges. Figure 7 shows the relationship between range difference and the baseline vector.

The range difference between $r_{A}^{k}$ (range from satellite *k* to Receiver A) and $r_{B}^{k}$ (range from satellite *k* to Receiver B) equal to the projection of the relative vector between Receiver A and Receiver B in the direction of the LOS of satellite *k*. The results are shown in Equation (12):

(12) $$r_{B}^{k}-r_{A}^{k}=h_{A}^{k}\left(X_{B}-X_{A}\right)$$

where, $X_{A}$ and $X_{B}$ are the receiver position in the navigation frame and $h_{A}^{k}$ is the LOS vector from satellite *k* to Receiver A.

The relative vector between two receivers can be represented by a baseline vector of Receiver A and Receiver B in Equation (1), and the rotation matrix $C_{b}^{n}$ is approximated as a function of the heading angle. The modified equation is as follows:

(13) $$r_{B}^{k}-r_{A}^{k}=h_{A}^{k}C_{b}^{n}\left(B_{B}-B_{A}\right)≃h_{A}^{k}R\left(\psi \right)\left(B_{B}-B_{A}\right)$$

where $B_{A}$ and $B_{B}$ are the baseline vectors from the reference point to Receiver A and Receiver B in the body frame, respectively and R(ψ) is the rotation matrix about heading angle ψ on the downward axis.

The double-difference pseudorange is modified by substituting Equation (13) into (11) as follows:

(14) $$\rho_{AB}^{jk}≃\left(h_{A}^{k}-h_{A}^{j}\right)R\left(\psi \right)\left(B_{B}-B_{A}\right)+\left(M_{B}^{k}-M_{A}^{k}\right)+\varepsilon_{\rho_{AB}^{jk}}$$

The first term of the light side is moved to the left side in Equation (14) to reduce the range difference in the double-difference pseudorange, and the remaining value is present the double difference of pseudorange error. It is difined as an error difference as follows:

(15) $$d_{AB}^{jk}=\rho_{AB}^{jk}-\left(h_{A}^{k}-h_{A}^{j}\right)R\left(\psi \right)B_{AB}^{}=\left(M_{B}^{k}-M_{A}^{k}\right)+\varepsilon_{\rho_{AB}^{jk}}$$

where $B_{AB}$ is the baseline vector between Receiver A and Receiver B in the body frame. The error difference includes the difference due to the bias error and noise between two receivers. If one receiver has a bias error or both receivers have bias errors that are significantly different, the error difference has a large value.

One way to use this value to detect the outliers is to use a hypothesis test. The statistical distribution of the error difference can be obtained from the statistical distribution of the pseudoranges that were calculated by Equation (9). Therefore, a significant error difference can be detected using the hypothesis test. As shown in Equation (15), a significant error difference can be caused by $M_{A}^{k}$ and $M_{B}^{k}$. Thus, it is difficult to determine which pseudorange is an erroneous one from the significant error difference, since both pseudorange could lead to a bias error.

Therefore, a closeness-based method is proposed and adapted instead of hypothesis test to detect outliers. This method distinguishes an erroneous pseudorange by comparing the size of the error difference from the same satellite. In particular, the mean of the error differences on any one receiver is calculated, and the distance of the error differences from the mean is computed for each error difference. The computed distance is defined as the closeness of measurements and is tested for outlier detection. If outliers exist, then the pseudorange with the largest distance is removed and the test is applied again for the remaining pseudoranges.

**Figure 8:**
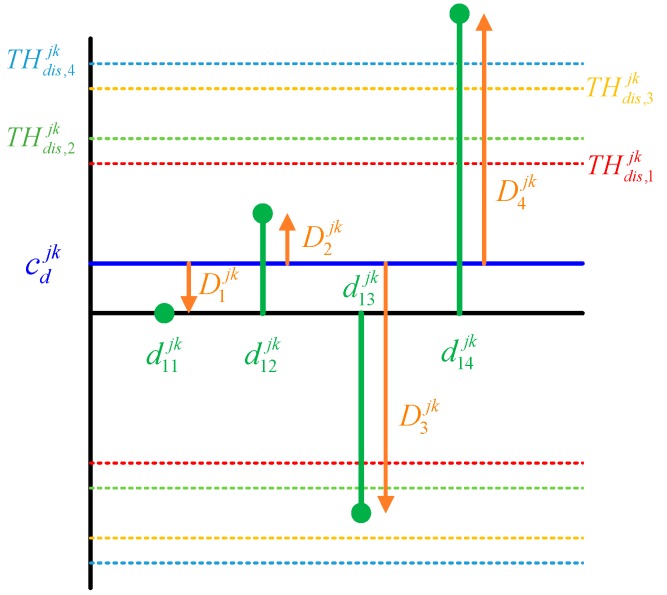
Example of the closeness test involving four receivers.

Figure 8 shows an example of the closeness test involving four receivers. In this example, the satellite *j* is selected as reference and the pseudoranges transmitted from satellite *k* are inspected. The error differences are calculated with respect to Receiver 1. The error differences, i.e., $d_{11}^{jk}$ to $d_{14}^{jk}$ are first calculated using Equation (15) as follows:

(16) $$d_{1s}^{jk}=\left(M_{s}^{k}-M_{1}^{k}\right)+\varepsilon_{\rho_{1s}^{jk}}$$

where *s* is the receiver number and has a value from 1, …, 4. Then, the mean of the error differences is calculated as follows:

(17) $$C_{d}^{jk}=\frac{1}{4}\left(d_{11}^{jk}+d_{12}^{jk}+d_{13}^{jk}+d_{14}^{jk}\right)$$

Since the error difference is calculated with respect to Receiver 1, the value of $d_{11}^{jk}$ is zero and Equation (17) is reduced to the following:

(18) $$C_{d}^{jk}=\frac{1}{4}\left(d_{12}^{jk}+d_{13}^{jk}+d_{14}^{jk}\right)$$

Finally, the distance from the mean of the error differences to each error difference is calculated and tested. The distance is defined as $D_{s}^{jk}$ and is calculated using Equation (19):

(19) $$D_{s}^{jk}=d_{rs}^{jk}-C_{d}^{jk}$$

Then each distance is obtained by substituting Equation (18) into Equation (19) as follows:

(20) $$\begin{array}{l} D_{1}^{jk}=-\frac{1}{4}\left(d_{12}^{jk}+d_{13}^{jk}+d_{14}^{jk}\right),\;D_{2}^{jk}=-\frac{1}{4}\left(d_{21}^{jk}+d_{23}^{jk}+d_{24}^{jk}\right)\\ D_{3}^{jk}=-\frac{1}{4}\left(d_{31}^{jk}+d_{32}^{jk}+d_{34}^{jk}\right),\;D_{4}^{jk}=-\frac{1}{4}\left(d_{41}^{jk}+d_{42}^{jk}+d_{43}^{jk}\right) \end{array}$$

Equation (20) shows that the distance for Receiver A is equal to the mean of the error differences with respect to Receiver A. If $D_{s}^{jk}$ is greater than the specified threshold, the relevant measurement is detected as an outlier. In this example, $D_{4}^{jk}$ exceeds the threshold and the pseudorange of Receiver 4 is detected as an outlier. This means that the pseudorange of Receiver 4 has bias error. Thus, each distance is represented as follows:

(21) $$\begin{array}{l} D_{1}^{jk}=-\frac{1}{4}\left[M_{4}^{k}+\varepsilon_{\rho_{12}^{jk}}+\varepsilon_{\rho_{13}^{jk}}+\varepsilon_{\rho_{14}^{jk}}\right],\;D_{2}^{jk}=-\frac{1}{4}\left[M_{4}^{k}+\varepsilon_{\rho_{21}^{jk}}+\varepsilon_{\rho_{22}^{jk}}+\varepsilon_{\rho_{24}^{jk}}\right]\\ D_{3}^{jk}=-\frac{1}{4}\left[M_{4}^{k}+\varepsilon_{\rho_{31}^{jk}}+\varepsilon_{\rho_{32}^{jk}}+\varepsilon_{\rho_{34}^{jk}}\right],\;D_{4}^{jk}=-\frac{1}{4}\left[-3M_{4}^{k}+\varepsilon_{\rho_{41}^{jk}}+\varepsilon_{\rho_{42}^{jk}}+\varepsilon_{\rho_{44}^{jk}}\right] \end{array}$$

As shown in Equation (21), the amplitude of the distance for Receiver 4 is approximately three times larger than that of the others. Thus, an erroneous pseudorange can be detected. If multiple pseudoranges with similar bias errors exist, the proposed method cannot detect the erroneous pseudoranges because the erroneous pseudorange cannot easily be distinguished from the distances. For example, if Receivers 1 and 2 have similar bias errors $M^{k}$, then each distance is calculated as follows:

(22) $$\begin{array}{ll} D_{1}^{jk}=-\frac{1}{4}\left[-2M^{k}+\varepsilon_{\rho_{12}^{jk}}+\varepsilon_{\rho_{13}^{jk}}+\varepsilon_{\rho_{14}^{jk}}\right], & D_{2}^{jk}=-\frac{1}{4}\left[-2M^{k}+\varepsilon_{\rho_{21}^{jk}}+\varepsilon_{\rho_{22}^{jk}}+\varepsilon_{\rho_{24}^{jk}}\right]\\ D_{3}^{jk}=-\frac{1}{4}\left[2M^{k}+\varepsilon_{\rho_{31}^{jk}}+\varepsilon_{\rho_{32}^{jk}}+\varepsilon_{\rho_{34}^{jk}}\right], & D_{4}^{jk}=-\frac{1}{4}\left[2M^{k}+\varepsilon_{\rho_{41}^{jk}}+\varepsilon_{\rho_{42}^{jk}}+\varepsilon_{\rho_{44}^{jk}}\right] \end{array}$$

The result shows that the amplitude of each of the distances is similar. Therefore, the proposed method can detect outliers only when the pseudoranges have a different bias error. If Receivers 1 and 2 have a different bias error $M_{1}^{k}>M_{2}^{k}$, then each distance is calculated as follows:

(23) $$\begin{array}{ll} D_{1}^{jk}=-\frac{1}{4}\left[M_{2}^{k}-3M_{1}^{k}+\varepsilon_{\rho_{12}^{jk}}+\varepsilon_{\rho_{13}^{jk}}+\varepsilon_{\rho_{14}^{jk}}\right], & D_{2}^{jk}=-\frac{1}{4}\left[M_{1}^{k}-3M_{2}^{k}+\varepsilon_{\rho_{21}^{jk}}+\varepsilon_{\rho_{22}^{jk}}+\varepsilon_{\rho_{24}^{jk}}\right]\\ D_{3}^{jk}=-\frac{1}{4}\left[M_{1}^{k}+M_{2}^{k}+\varepsilon_{\rho_{31}^{jk}}+\varepsilon_{\rho_{32}^{jk}}+\varepsilon_{\rho_{34}^{jk}}\right], & D_{4}^{jk}=-\frac{1}{4}\left[M_{1}^{k}+M_{2}^{k}+\varepsilon_{\rho_{41}^{jk}}+\varepsilon_{\rho_{42}^{jk}}+\varepsilon_{\rho_{44}^{jk}}\right] \end{array}$$

In this case, $D_{1}^{jk}$ has the largest amplitude and the pseudorange of Receiver 1 is detected as an outlier and is removed. The test is applied again for the remaining Receivers 2, 3, and 4. In the second test, Receiver 2 is detected as an outlier and removed. As a result, two outliers are detected and removed by the closeness test.

If no bias error exists for all the pseudoranges, then the distance consists of double differenced noise error as shown in Equation (15). The distance threshold is obtained by calculating the theoretical double-differenced noise error which is computed using Equation (9) and the SNR measurements as follows:

(24) $$\varepsilon_{\rho_{AB}^{jk}}=\sqrt{{\left(\sigma_{A}^{j}\right)}^{2}+{\left(\sigma_{A}^{k}\right)}^{2}+{\left(\sigma_{B}^{j}\right)}^{2}+{\left(\sigma_{B}^{k}\right)}^{2}}$$

To prevent a high probability of miss detection, the higher limit of the distance threshold is applied. This limit is determined from the maximum pseudorange noise error. In this research, 1 m is empirically selected as the value of the maximum pseudorange noise error. The distance threshold for pseudorange *s* is calculated using the following equation:

(25) $$TH_{Dis,\;s}^{jk}=3\times \min\left(\sum_{g=1}^{N}\varepsilon_{\rho_{sg}^{jk}},\;2N\cdot \sigma_{\max}\right)$$

where *N* is the number of pseudoranges and $\sigma_{\max}$ is the maximum pseudorange noise error, which depends on the receiver used. The threshold is changed according to the pseudoranges that are used, the number of pseudoranges, and the pseudorange that has yet to be tested.

**Figure 9:**
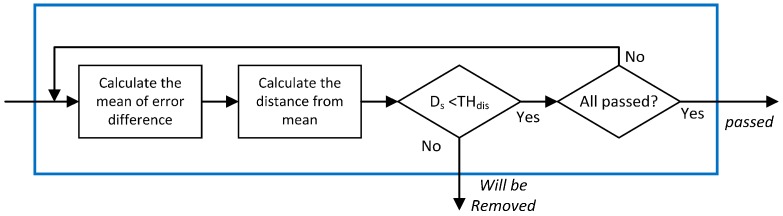
Flowchart of closeness test.

The flowchart of the closeness test is shown in Figure 9. After rejecting the outlier, the mean of error differences is recalculated using the remaining error differences, and the distance from the mean is checked again. If all measurements are less than the threshold, they are passed to the next stage.

**Figure 10:**
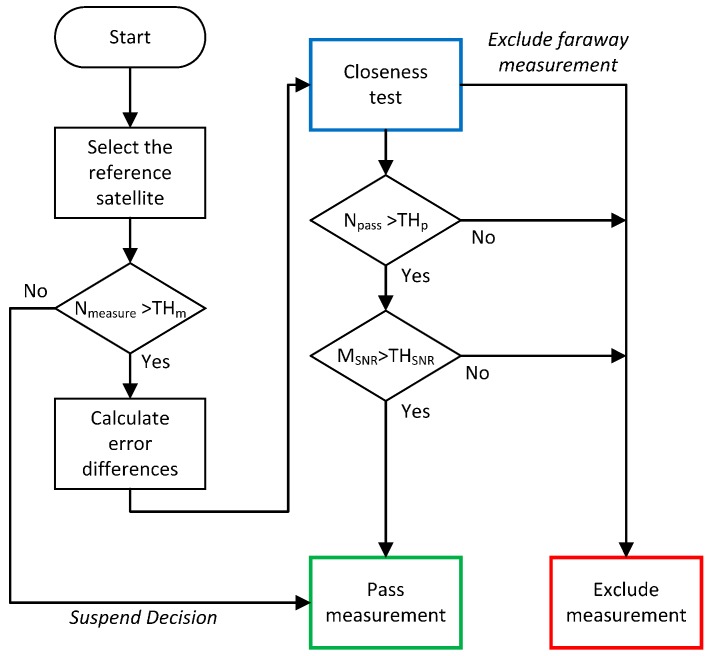
Overall flowchart of outlier detection method.

The overall flowchart of the proposed outlier detection algorithm for pseudoranges transmitted from the same satellite is presented in Figure 10. To detect outliers, the redundancy should be sufficient. Thus, the number of measurements is checked before applying the closeness test, and the minimum number of measurement is defined as *TH*_m_. To apply the closeness test, the number of measurement should be greater than two. If this condition is not satisfied, the test is suspended to the next stage, i.e., RAIM algorithm. Following the closeness test, the number of passed measurements is checked, which should be greater than the threshold *TH*_p_. This value depends on the number of receivers, and is determined empirically. In this paper, we assumed two for four multiple receivers. Although, the pseudoranges pass the closeness test, those can be outliers when common bias is included in most pseudoranges. To separate these measurements, an SNR test is performed because it is known that multipath or NLOS measurements have lower SNR values than direct measurements [10]. If the mean SNR of the passed measurements is lower than the threshold, those are regarded as outliers and are rejected. The threshold *TH*_SNR_ depends on the type of receivers, and is determined empirically. In this study, *TH*_SNR_ is selected as 35 dB.

## 4. Experiment

### 4.1. Experimental Environment

**Figure 11:**
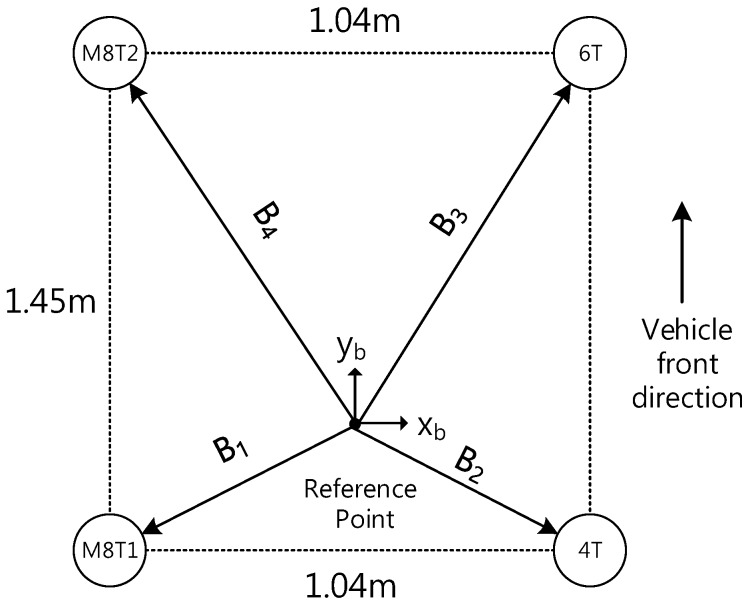
Antenna configuration and locations.

Four receivers were used for the experiment to test the proposed method. They were installed along the edge of the roof of a vehicle. The distance among the receivers was limited to 1–2 m due to the size of the vehicle. Each multi-receiver consisted of patch antennas and low-cost U-blox receivers: two EVK-M8T, one EVK-6T and one AEK-4T. Figure 11 shows the configuration and location of antennas on the roof of the vehicle. The Novatel GPS RTK/INS integration system (SPAN) was used as a reference sensor to evaluate the positioning performance. The antenna of SPAN was also mounted on the reference point of the roof as shown in Figure 11. Measurements of multiple receivers were logged at a rate of 1 Hz and was post-processed using the proposed method.

**Figure 12:**
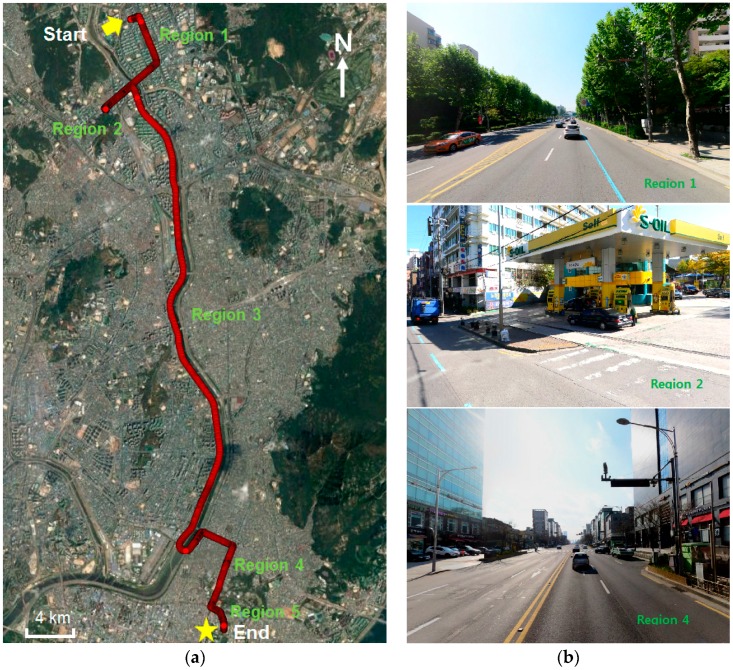
Vehicle trajectory for the test. (**a**) Ground truth; (**b**) Images along vehicle trajectory (**Top**: Region 1, high buildings and tree; **Middle**: Region 2, gas station; **Bottom**: Region 4, high buildings).

The test was carried out by driving the vehicle from Junggye station to Konkuk University in Seoul, Korea for a total of approximately 13.7 km. Figure 12 shows the trajectory of the vehicle and images along the route. In Region 1, buildings and trees prevented the propagation of the GPS signal, and the road was lined by high buildings in Region 4, as shown in Figure 12b. The proposed method was tested under stationary conditions by stopping the vehicle at a gas station and by collecting data for approximately 10 min as shown in the figure in the center of Figure 12b. Especially in this area, the positioning performance was degraded in the RAIM algorithm as well as general positioning algorithm due to multiple outliers. Thus, position performance did not improve to a significant degree. The vehicle was moved onto a motorway in Region 3 to test the proposed method in an open sky area. In Region 5, the road was flanked by buildings and trees.

The vehicle’s heading angle used in the proposed algorithm was obtained by adding the noise, as mentioned before. In practice, the noise in the calculation of the heading angle from a two-antenna GPS system with a 1-m baseline was 0.1° in the open sky [25]. For unfavorable signal environments, the standard deviation of added noise was determined to be 5°. The maximum value of noise with standard deviation of 5° is about 20° (4-sigma), and lead to an error of approximately 0.6 m when rotating the baseline vector from the body frame to the navigation frame.

In this paper, three methods of outlier detection were used, and their positioning performance compared. Method 1 represents the general measurement test among positioning algorithms. Method 2 is the RAIM algorithm for multiple receivers explained in Section 3. The proposed method was named Method 3.

- Method 1: Elevation Angle > 15°, SNR > 35 dB
- Method 2: Method 1 + RAIM algorithm
- Method 3: Method 2 + Pseudorange comparison

### 4.2. Experimental Results

**Figure 13:**
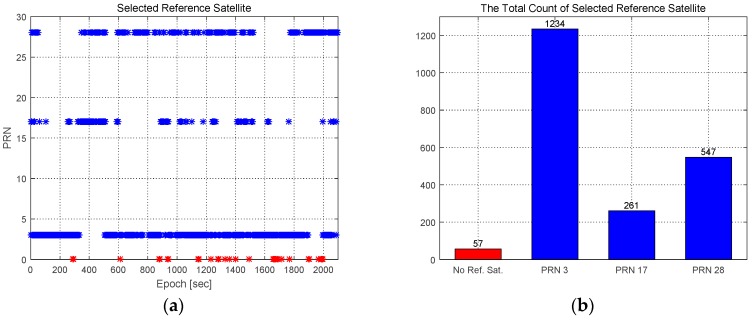
Results of the selection of the reference satellite. (**a**) The chosen PRN number; (**b**) Total number of selected reference satellites.

**Figure 14:**
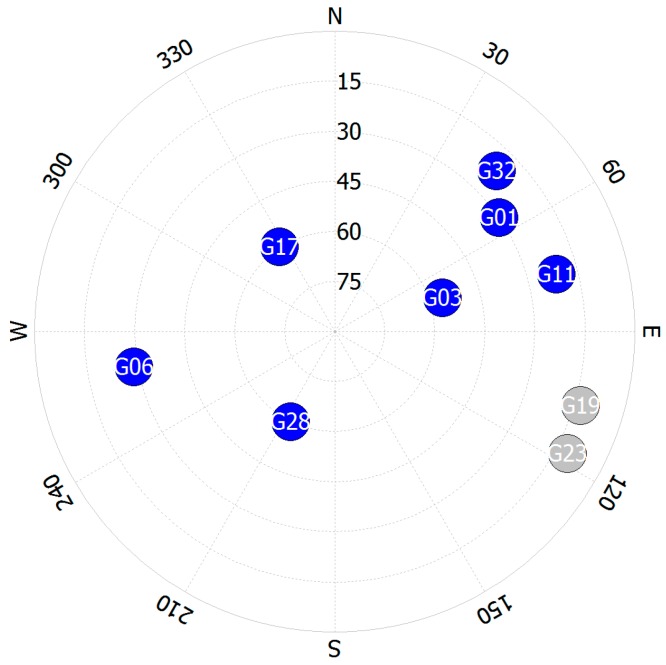
Skyplot of the observed satellites during the experiment.

Prior to analyzing performance, the selection results of the reference satellite are presented. Figure 13 shows the Pseudo Random Noise (PRN) number and the total number of selected reference satellites. PRN 3 is mostly selected for the reference satellite, as shown in Figure 13b. The skyplot of observed satellites during the experiment is shown in Figure 14. The blue color of the PRN number represents satellites with higher elevation angles than the cutoff angle. PRN 3, PRN 17, and PRN 28 had high elevation angles. When a reference satellite did not exist, the selected reference satellite set the PRN number to zero, and is shown as a red point in Figure 13a. The total absence of a reference satellite is 57 epochs, and only 2.7% of the total number of epochs. This was a reasonable value in an urban area, and the assumption of the existence of a reference satellite was appropriate. If multiple GNSSs could be jointly used, the reference satellite would exist most of the time. Therefore, the proposed outlier detection method can be applied to every epoch.

**Figure 15:**
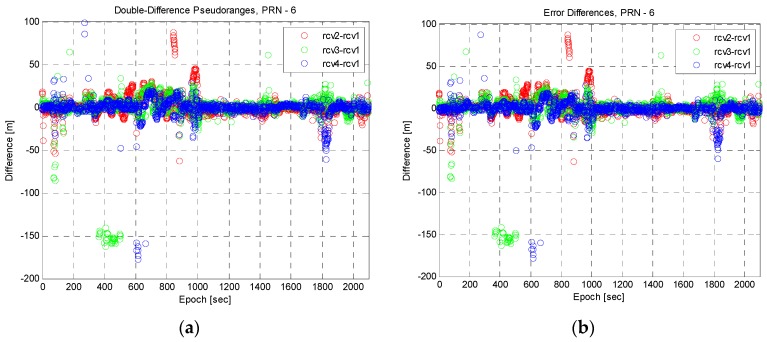
Pre-processing results of pseudoranges. (**a**) Double-difference pseudoranges; (**b**) Error differences represented in Equation (15).

The pre-processing results of pseudoranges for PRN 6 are presented in Figure 15. The double-difference pseudoranges are shown in Figure 15a, and Receiver 1 is used as the reference receiver. Figure 15b shows the error differences already defined in Equation (15). The difference between any two results did not seem to be significant because the distance between multiple receivers was short. If the bias error was not included in the error difference, it was modeled as double-difference noise with a normal distribution as computed in Equation (24). However, the bias errors caused a significant in error difference as shown in Figure 15b, especially between epochs 400 and 600 s. Using this property, the error difference can be used to check the existence of outliers, but it is difficult to identify them.

**Figure 16:**
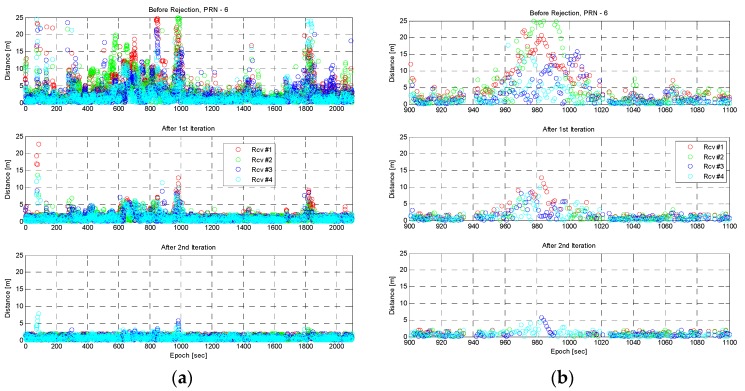
The distance from the mean of error differences with closeness test for PRN 6. (**a**) All epochs; (**b**) Epochs between 900 and 1100 s. **Top**: before closeness test; **Middle**: after first iteration; **Bottom**: after second iteration.

To detect and identify outliers, the closeness test was applied to the error differences. Figure 16 shows the result of the closeness test. First, the mean of error differences was calculated, the results of which are shown at the top of Figure 16a. Then maximum distance was compared with a threshold; if it was greater than the threshold, it was rejected. Following the rejection of a measurement larger than the threshold, the distance from a mean was calculated again, as shown at the middle of Figure 16a. The process was repeated to remove the remaining large value. As shown at the bottom of Figure 16a, almost all large values were removed after the second iteration. Figure 16b shows the results between epochs 900 and 1100 s. The pseudoranges of Receiver 1, 2, and 3 were removed following the closeness test, around epoch 980 s.

**Figure 17:**
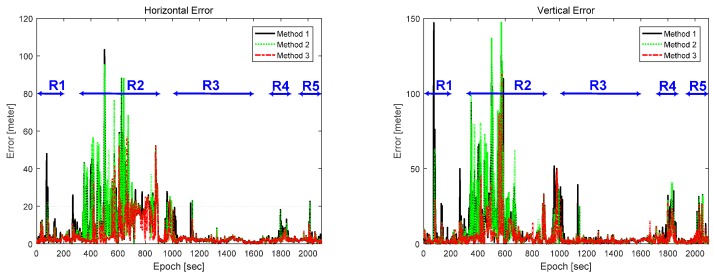
Position errors: horizontal (**Left**) and vertical (**Right**).

The performance of the three methods was evaluated and analyzed by calculating the horizontal and vertical positioning errors which are graphically depicted in Figure 17. The test regions are presented as R1~R5. The error was calculated based on the Novatel SPAN & Inertial Explorer (Post-processing of CDGPS/IMU Integration). As shown in Figure 17, Method 3 had smaller positioning errors than the other methods in almost all epochs. In Regions 1, 4, and 5, buildings and trees cause multipath and NLOS errors and the positioning performance is degraded in Methods 1 and 2. In the open sky area, i.e., Region 3, three of the methods show the similar positioning accuracy. In Region 2, as mentioned before, the vehicle stopped at a gas station, thereby causing the position accuracy to be degraded for all the methods. Nonetheless, Method 3 improved the positioning accuracy significantly. Although Method 2 also improved the positioning accuracy, the improvement was insufficient. In Region 2, the vehicle is moved by a small displacement and the positioning accuracy is changed at around epoch 500 and 600 s.

**Figure 18:**
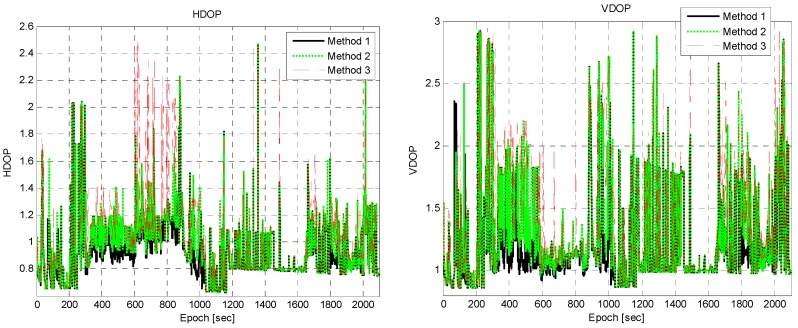
HDOP (**Left**) and VDOP (**Right**).

**Figure 19:**
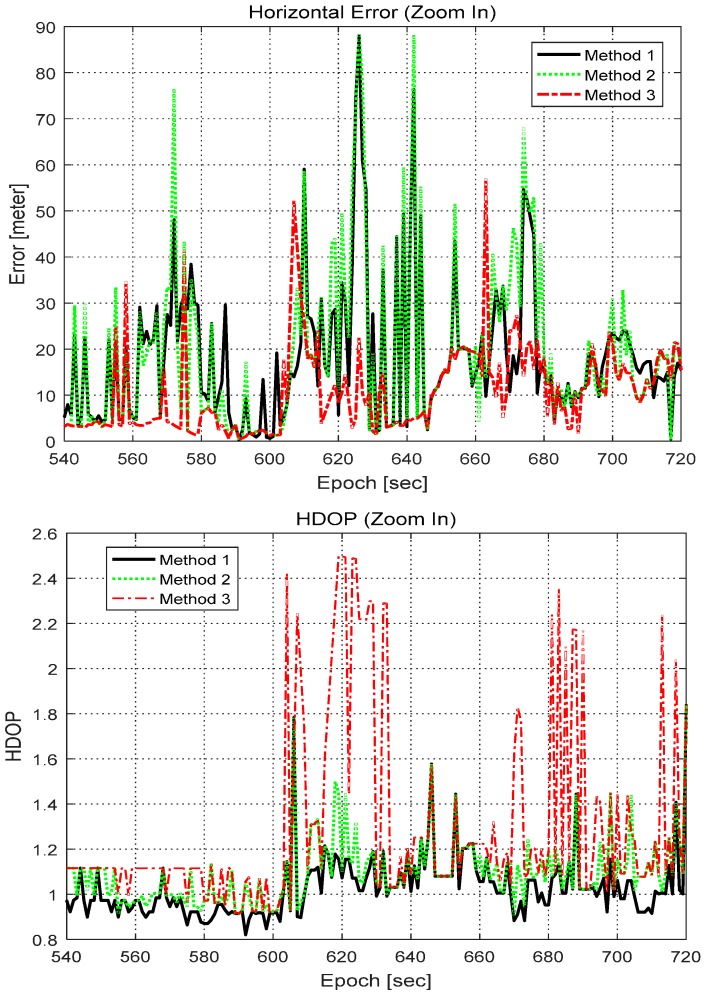
Expended plot of horizontal error (**Top**) and HDOP (**Bottom**).

In some epochs, such as 558, 608, 663, 877, and 979 s, even Method 3 recorded a larger error than Method 1. To investigate the reason for this, the dilution of precision (DOP) of the three methods was calculated, and is shown in Figure 18. Since Method 3 rejected many measurements, it had larger DOPs than the other two methods. The expanded plot of the horizontal error and HDOP between epochs 540 and 720 s is shown in Figure 19. In epoch 608 s, the horizontal error of Method 3 was significant, and its HDOP was similarly large. The other epochs such as 558 and 663 s cannot be explained in terms of the increase in DOP.

**Figure 20:**
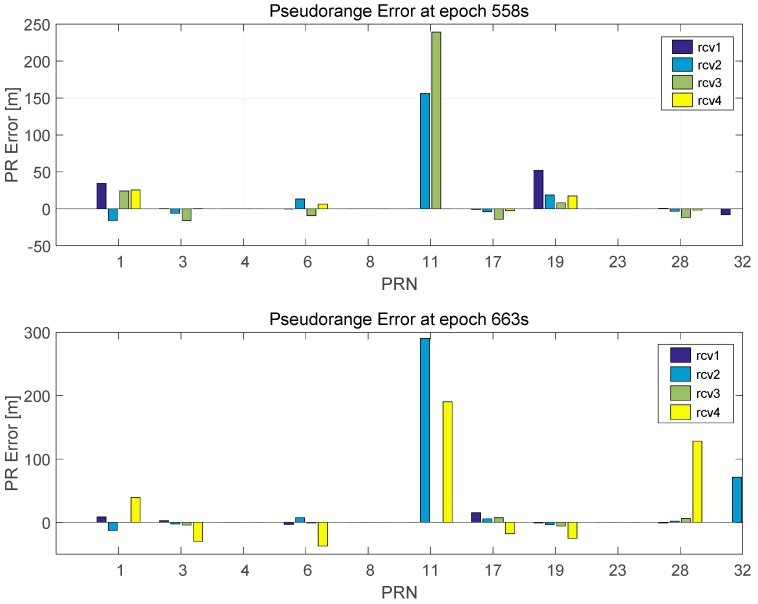
Pseudoranges error at epoch 558 s (**Top**) and 663s (**Bottom**).

The reason was investigated by representing the pseudorange errors of these epochs in Figure 20. The pseudorange errors are calculated by estimating the receiver clock error using the position of the reference sensor. At epoch 558 s, PRN 11 has two pseudoranges with a large error as shown at the top of Figure 20. The pseudorange comparison method cannot be applied since the number of pseudoranges for PRN 11 does not exceed the threshold. Thus, the erroneous pseudoranges are suspended and tested in the RAIM algorithm. However, the large pseudorange error prevents the operation of the RAIM algorithm and the positioning error is increased.

The error in the pseudoranges at epoch 663 is presented at the bottom of Figure 20. In this epoch, PRN 3 is selected as reference satellite. However, the pseudorange of Receiver 4 for PRN 3 contains a large error and the closeness test cannot detect the erroneous pseudoranges.

**Figure 21:**
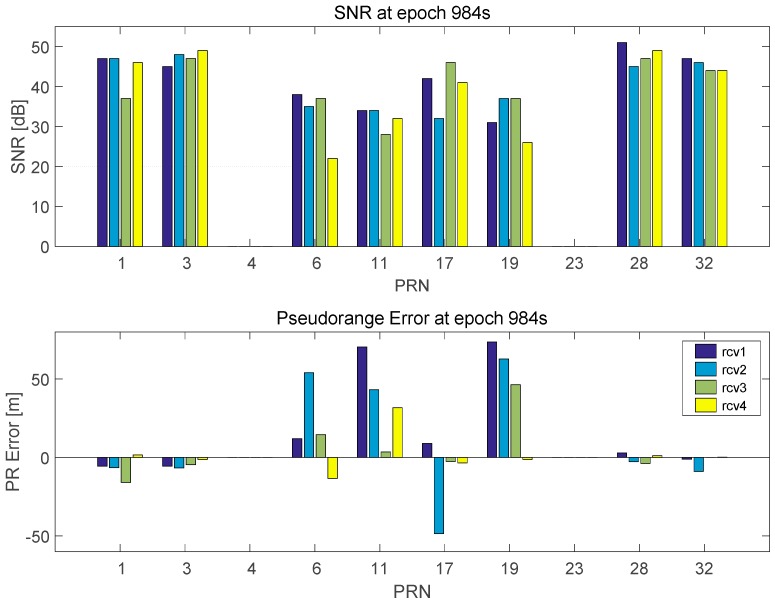
SNRs (**Top**) and pseudoranges (**Bottom**) error at 984 s.

Moreover, the position error is not improved at epoch 877 s because the reference satellite did not exist and thus the pseudorange comparison method cannot be applied. In epoch 984 s, the common and large bias error in the pseudoranges prevents the detection of outliers. Figure 21 shows the SNRs and the pseudorange errors at this epoch. The closeness test can detect the erroneous pseudoranges in PRN 6, 11, and 17; however, PRN 19 cannot be detected since the pseudoranges in PRN 19 have a common bias error as shown in Figure 21. Even the inlier in the pseudorange, i.e., measured in Receiver 4, is incorrectly detected as an outlier and removed. Furthermore, the SNR test fails because the mean SNR of PRN 19 measured in Receivers 1–3 is larger than the SNR threshold (35 dB).

In Section 3.2, the common bias error of the pseudoranges was considered to be the main weakness of the proposed method. Since the SNR test can detect pseudoranges with a common bias, this does not affect the positioning performance as expected. In addition, as mentioned in Section 1, the measurements obtained in urban areas from multiple receivers with a short baseline can have different error characteristics [19]. As a result, Method 3 provided excellent outlier detection ability and improved the positioning accuracy. However, this method sometimes led to large positioning errors due to an increased DOP, a large pseudorange error in the reference satellite, and common bias error. Furthermore, the proposed pseudorange comparison method cannot be applied when a reference satellite does not exist and insufficient pseudoranges are measured.

Table 1 represents positioning errors in terms of mean, standard deviation, and maximum. These values were calculated using all available epochs of each method. Method 3 had smaller errors than the other two methods for all parameters. Methods 2 and 3 improved horizontal accuracy by approximately 7% and 41% with respect to Method 1, respectively. Vertical accuracy was enhanced by approximately 13% and 53% for Methods 2 and 3, respectively. Similarly, Method 3 improved horizontal and vertical accuracy by 36% and 46% compared to Method 2, respectively. Also, the availability was represented in Table 1 and was calculated by ratio of available epochs to all epochs. The result shows that the proposed method has smallest availability than other method but the difference is small and acceptable.

**Figure 22:**
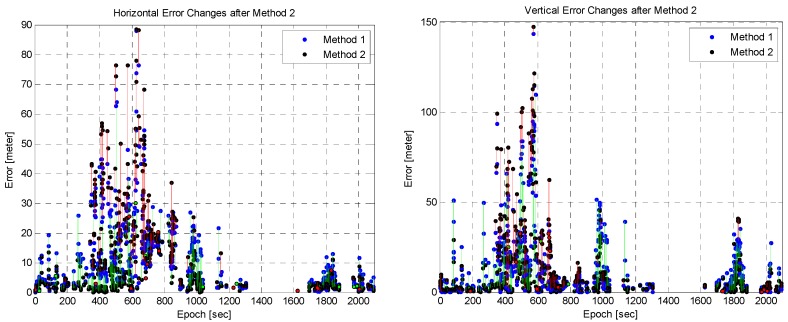
Position error changes with Method 2: horizontal (**Left**) and vertical (**Right**).

**Figure 23:**
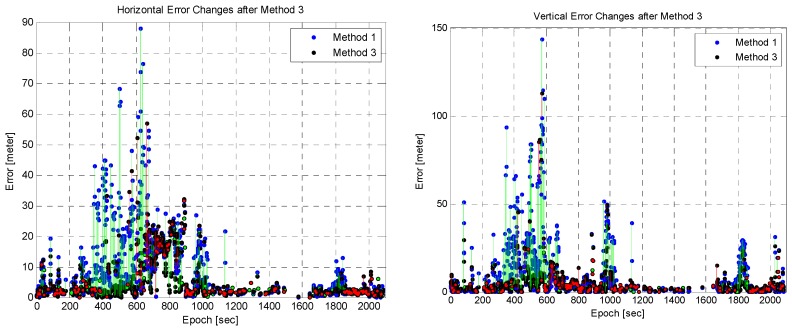
Position error changes with Method 3: horizontal (**Left**) and vertical (**Right**).

In order to further analyze the performance of Methods 2 and 3, the changes in their position errors were computed and compared. As explained before, Methods 2 and 3 were applied in addition to Method 1 for outlier detection. Thus, the changes in position errors following outlier detection were evaluated. The changes in the position error after Methods 2 and 3 are shown in Figure 22 and Figure 23, respectively. The blue and black points represent the position error of Method 1 and those of Methods 2 and 3, respectively. Changes in the position error are indicated by a line connecting the two points. An improvement in the position error is indicated by a green line; otherwise red line is used. As shown in Figure 22 and Figure 23, considerably more position errors were reduced for Method 3 than for Method 2. Many of these were reduced by a large amount, and many others increased by a small amount. The results are summarized in Table 2, where the number in parentheses indicates the total number of changes. Method 3 reduced the position error by a large amount than Method 2, but the number of position errors that increased is larger than for Method 2. This means that Method 3 has a higher probability of fault detection than Method 2. In Method 3, outlier detection was performed for each satellite measurement from only one satellite. This caused a high probability of fault detection. Thus, the use of additional receivers can reduce fault detection. In spite of the high fault detection probability, the amount by which the error of Method 3 increased is smaller than that of Method 2. This implies the superiority of the proposed method.

**Figure 24:**
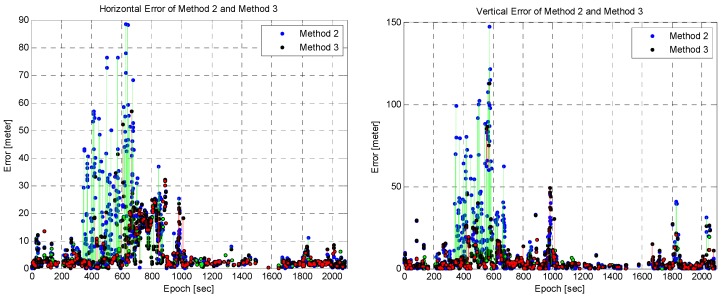
Position error of Method 2 and Method 3: horizontal (**Left**) and vertical (**Right**).

To compare the position changes by the proposed method, the position errors of Method 2 and 3 are represented in Figure 24. In the area in which the vehicle stopped, the position accuracy is improved significantly by Method 3. In this area, the RAIM algorithm cannot detect the outliers because the large position error prevents the operation of the RAIM algorithm. Moreover, this means that the four receivers had a different bias error for each of their pseudoranges and that Method 3 can detect the outliers. The difference in the bias errors was caused by the closed wall and roof of the gas station. Around epoch 980 s, the position error in both the horizontal and vertical directions of Method 3 increases. As mentioned above, these results are caused by the common bias error in the pseudoranges and the failure of the SNR test.

**Figure 25:**
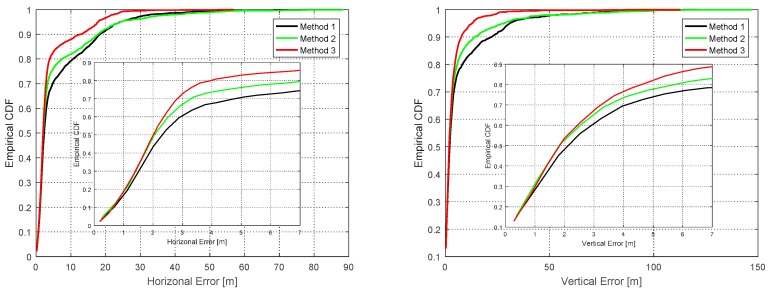
CDF of horizontal (**Left**) and vertical error (**Right**).

In order to evaluate the distribution of position error, the cumulative density functions (CDF) of the position errors were computed, and the results are shown in Figure 25. The inner box represents the expanded plot epochs between 0 to 7 s. The red and green lines crossed at a vertical error of 1.5 m. Method 2 had a higher value within 1.5 m of vertical error, and Method 3 greater than above it. As mentioned before, the high probability of fault detection of Method 3 caused this result. However, the differences with 1.5 m of vertical error were very small, and this error was the general level of error for low-cost GPS receivers. Method 3 could detect more significant errors than Method 2, and hence improved the overall positioning performance.

**Figure 26:**
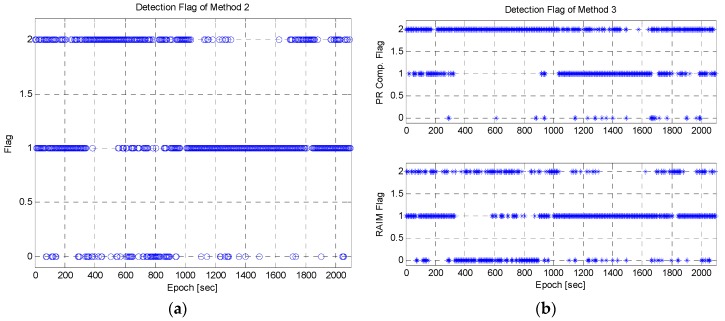
The detection flag: (**a**) method 2; (**b**) method 3. The flag was set to zero when it was not possible to test, where 1 meant that the test was applied but outlier was not detected, and 2 implied that the test was applied and the outlier was detected.

To analyze the extent of cooperation between the pseudorange comparison method and the RAIM algorithm, the detection flag is shown in Figure 26. Figure 26a,b show the detection flag of Method 2 and Method 3, respectively. The detection epochs of the RAIM algorithm for Method 3 were smaller than those of Method 2. In Method 3, the pseudorange comparison method was applied ahead of the RAIM algorithm, and passed and suspended measurements were tested again with the RAIM algorithm. Thus, the number of cases of detection by the RAIM algorithm was reduced. However, the pseudorange comparison method detected outliers in many epochs. When the pseudorange method missed outliers, the RAIM algorithm detected the remaining ones. For example, the remaining outliers were detected in the epoch between 300 and 600 s as shown at the bottom of Figure 26b. However, the detection case for the pseudorange comparison method (flag 2 at the top of Figure 26b) was much more than the RAIM algorithm (flag 2 at the bottom of Figure 26b) in the between 300 and 1000 s. It implies that the most improvements of position accuracy were caused by the pseudorange comparison method. Actually, the number of detection case for the RAIM algorithm was only 644 for all epochs and for the proposed algorithm 1197.

## 5. Conclusions

In this paper, a positioning algorithm for multiple receivers was proposed to enhance positioning performance in urban areas. The multiple receivers were integrated based on a tightly coupled approach. In order to remove outliers such as the multipath and NLOS errors, the pseudorange comparison method was proposed for pseudoranges from the same satellite. Before applying the closeness test, non-common terms were removed by double-differencing the pseudoranges and projecting the baseline vector of multiple receivers. To reduce the fault detection, an SNR test was applied after the closeness test. In order to analyze the performance of the proposed algorithm, a land vehicle test was performed in an urban area. The results showed that outlier detection is enhanced by the pseudorange comparison method, and position performance hence improves. The position accuracy is enhanced about 36% for horizontal and about 46% for vertical compared to the conventional RAIM algorithm. As a result, the test results show the feasibility and effectiveness of the proposed method. The advantages and drawbacks of the proposed method are summarized below:

*Advantages*: First, this approach can check outliers without calculating position, and the mutual influence of measurements does not affect outlier detection. Therefore, it has better outlier detection capability for multiple or large outliers than the RAIM-based method. Second, since the proposed algorithm is applied prior to the RAIM algorithm, it can be used together with the latter. Third, multiple receiver systems can be implemented easily, and are inexpensive. Actually, the proposed method is almost simplest and cheapest method among the previously proposed methods. Furthermore, the positioning algorithm involving a single receiver can be used with the proposed method with minor modifications.

*Drawbacks*: First, when the common bias is included in pseudoranges, the closeness test cannot detect the outliers. The affection of common bias can be reduced by the SNR test and the test results shows that the common bias does not exist in many epochs; Second, the proposed method only uses pseudoranges from the same satellite, and the consequent lack of information leads to a high probability of faulty detection. The rejection of inlier measurement increases the DOP and the position error. To overcome this problem, additional information is needed such as more multiple receivers or assistance from other satellites; Third, in order to apply this method, the reference satellite is needed, and sufficient measurements must be obtained. The joint use of multiple GNSSs can solve the reference satellite problem, and the use of more multiple receivers can enhance the number of measurement.

*Future Work*: First, the passed pseudoranges can have a common bias, and these are rejected by checking the SNR in the closeness test. However, if the bias is common, it could be estimated in the positioning algorithm by adding one unknown variable to the state vector. To check its feasibility, the observability analysis and live data test will be performed; Second, accurate measurement of the attitude or the heading angle can improve the proposed method. Therefore, an attitude estimation method using carrier phase measurement from multiple receivers will be implemented in future work.

## Figures and Tables

**Table 1:** Position errors and Availability.

Methods	Horizontal Errors (m)			Vertical Errors (m)			Availability (%)
	Mean	Std	Max	Mean	Std	Max	
**Method 1**	7.04	10.29	103.2	8.05	14.91	146.97	97.9
**Method 2**	6.61	10.89	95.51	6.97	14.71	147.61	97.6
**Method 3**	4.18	5.81	57.03	3.79	6.34	113.12	94.8

**Table 2:** Position error changes with outlier detection and exclusion.

Methods	Total Amount of Horizontal Error Changes (m)		Total Amount of Vertical Error Changes (m)	
	Reduced	Increased	Reduced	Increased
**Method 2**	2018.0 (407)	1217.9 (237)	3807.6 (383)	1933.6 (261)
**Method 3**	5123.6 (784)	478.1 (413)	6875.8 (784)	632.2 (462)
**Method 3 w.r.t. Method 2**	4385.1 (722)	462.3 (475)	5026.5 (722)	691.6 (520)
